# Modelled biophysical impacts of conservation agriculture on local climates

**DOI:** 10.1111/gcb.14362

**Published:** 2018-07-18

**Authors:** Annette L. Hirsch, Reinhard Prestele, Edouard L. Davin, Sonia I. Seneviratne, Wim Thiery, Peter H. Verburg

**Affiliations:** ^1^ Institute for Atmospheric and Climate Science ETH Zurich Zurich Switzerland; ^2^ Environmental Geography Group Institute for Environmental Studies Vrije Universiteit Amsterdam Amsterdam The Netherlands; ^3^ Department of Hydrology and Hydraulic Engineering Vrije Universiteit Brussel Brussels Belgium; ^4^ Swiss Federal Research Institute WSL Birmensdorf Switzerland

**Keywords:** CESM, climate‐effective land management, CLM, land‐based mitigation, subgrid‐scale influences, temperature extremes, tillage

## Abstract

Including the parameterization of land management practices into Earth System Models has been shown to influence the simulation of regional climates, particularly for temperature extremes. However, recent model development has focused on implementing irrigation where other land management practices such as conservation agriculture (CA) has been limited due to the lack of global spatially explicit datasets describing where this form of management is practiced. Here, we implement a representation of CA into the Community Earth System Model and show that the quality of simulated surface energy fluxes improves when including more information on how agricultural land is managed. We also compare the climate response at the subgrid scale where CA is applied. We find that CA generally contributes to local cooling (~1°C) of hot temperature extremes in mid‐latitude regions where it is practiced, while over tropical locations CA contributes to local warming (~1°C) due to changes in evapotranspiration dominating the effects of enhanced surface albedo. In particular, changes in the partitioning of evapotranspiration between soil evaporation and transpiration are critical for the sign of the temperature change: a cooling occurs only when the soil moisture retention and associated enhanced transpiration is sufficient to offset the warming from reduced soil evaporation. Finally, we examine the climate change mitigation potential of CA by comparing a simulation with present‐day CA extent to a simulation where CA is expanded to all suitable crop areas. Here, our results indicate that while the local temperature response to CA is considerable cooling (>2°C), the grid‐scale changes in climate are counteractive due to negative atmospheric feedbacks. Overall, our results underline that CA has a nonnegligible impact on the local climate and that it should therefore be considered in future climate projections.

## 1 INTRODUCTION

Agricultural land management has a substantial impact on regional climate (e.g. Davin, Seneviratne, Ciais, Olioso, & Wang, 2014; Hirsch, Wilhelm, Davin, Thiery, & Seneviratne, 2017; Luyssaert et al., 2014; Thiery et al., 2017), and influences local responses to projected climate change (Hirsch et al., 2017). However, historically climate model simulations contributing to the various Coupled Model Intercomparison Projects (CMIPs) have had limited or no representation of agricultural land management. Recently, considerable progress has been made to move away from basic representations of agricultural activity in Earth System Models (ESMs) where crops are typically modelled with the same physiological characteristics as C3 grasses. This includes the parameterization of irrigation (e.g. Cook, Shukla, Puma, & Nazarenko, 2014; De Vrese, Hagemann, & Claussen, 2016; Decker, Ma, & Pitman, 2017; Guimberteau, Laval, Perrier, & Polcher, 2012; Harding & Snyder, 2012; Lawston, Santanello, Zaitchik, & Rodell, 2015; Qian, Huang, Yang, & Berg, 2013; Sacks, Cook, Buenning, Levis, & Helkowski, 2008; Thiery et al., 2017) to fully interactive crop modules that incorporate different crop varieties with defined growing seasons and fallow periods (e.g. Levis et al., 2012).

However, conservation agriculture, involving minimal or no tillage, crop residue management and crop rotation (Kassam, Friedrich, Derpsch, & Kienzle, 2015), is generally not considered in the land surface component of ESMs. This is due to (a) a lack of global datasets characterizing where conservation agriculture is practiced and (b) the high uncertainty in soil carbon sequestration under minimal tillage regimes (Neufeldt, Kissinger, & Alcamo, 2015; Powlson et al., 2014). Nevertheless, the adoption of these land management practices is seen as a potential climate mitigation and adaptation strategy that also has climate impacts beyond its impact on carbon sequestration (Davin et al., 2014; Lobell, Bala, & Duffy, 2006). Here, we take the first step towards resolving the representation of conservation agriculture within ESMs by implementing a simple parameterization within a state‐of‐the‐art ESM using a new spatially explicit dataset on conservation agriculture as input (Prestele, Hirsch, Davin, Seneviratne, & Verburg, 2018).

The adoption of conservation agriculture has increased from 45 Mha in 1999 to 157 Mha in 2013 (Derpsch, Friedrich, Kassam, & Hongwen, 2010; Kassam et al., 2015). This increase is partly associated with the need to increase productivity in response to various external economic and environmental pressures (Kassam et al., 2015). Furthermore, this increase is influenced by the growing awareness of how traditional tillage‐based production systems have a negative impact on soil quality by increasing soil erosion rates and changing biogeochemical cycling due to soil disturbance (Govers, Quine, Desmet, & Walling, 1996; Govers, Vandaele, Desmet, Poesen, & Bunte, 1994; Quinton, Govers, Van Oost, & Bardgett, 2010; Van Oost et al., 2007; Wang et al., 2017) which has potential negative consequences for yield. In particular, yield levels with minimal tillage are comparable or even higher than conventional tillage systems (Kassam et al., 2015). Nonetheless, a recent meta‐analysis does identify that yield gains from minimal tillage are mostly possible when combined with both crop residue management and crop rotation, particularly for drier climates rather than humid climates (Pittelkow et al., 2015). There are, however, some critical limitations on where conservation agriculture is possible. This includes practical knowledge about conservation agriculture and investment costs—including time and machinery—to establish and maintain the soil mulch, and finally the political and social support to move away from traditional farming practices (Kassam et al., 2015). Furthermore, conservation agriculture has biophysical feedbacks on climate associated with how the presence of a crop residue alters the surface energy balance through changes in surface albedo, roughness, and evapotranspiration (Davin et al., 2014).

The examination of the potential impacts of no‐till farming, an essential part of conservation agriculture, on the climate system has been done in climate models. However, existing studies often use an idealized approach to evaluate the climate implications of a full conversion of global croplands (e.g. Davin et al., 2014; Hirsch et al., 2017; Lobell et al., 2006; Wilhelm, Davin, & Seneviratne, 2015). For example, Lobell et al. (2006) compare the impact of four different land management practices on present‐day climate. In that study, no‐till farming was represented by multiplying the soil albedo by 1.5 over the fractional area designated as croplands in the model. The results demonstrated the potential of increasing surface albedo to cool surface temperatures by reducing the available energy at the surface. Davin et al. (2014) apply more conservative changes over Europe in a regional climate model by increasing surface albedo over croplands by 0.1 and increasing the soil resistance by a factor of 4 to represent the effects of crop residue on evaporation. They found that the cooling potential of no‐till farming was greater for temperature extremes than mean temperature. Wilhelm et al. (2015) also take an idealized approach to represent no‐till via albedo changes and demonstrate that the temperature response scales linearly with the magnitude of the albedo change, the spatial extent, and the temporal extent. This was confirmed in Hirsch et al. (2017) who also demonstrate that the cooling potential from increased surface albedo associated with no tillage is comparable to that of irrigation, and therefore the effects of surface albedo changes associated with agricultural practices are important. Although these studies (i.e. Davin et al., 2014; Hirsch et al., 2017; Lobell et al., 2006; Wilhelm et al., 2015) use an idealized approach to examine the cooling potential of various land management practices, they all demonstrate that changes in albedo and evapotranspiration associated with no‐till farming have implications for climate, particularly temperature extremes, and that therefore investment in further developing parameterizations to represent this land management practice, and more specifically conservation agriculture, within ESMs is worth pursuing.

In this study, we build upon previous research with ESMs to examine the climate implications of agricultural land management. Deviating from the idealized approach used in previous studies, we use a new global conservation agriculture dataset (Prestele et al., 2018) to constrain the application of albedo and evapotranspiration changes towards a more realistic distribution of this land management practice. We also limit albedo changes to the soil surface rather than the canopy albedo as in Wilhelm et al. (2015) and Hirsch et al. (2017) to emulate how the presence of crop residues alters the background surface albedo. Furthermore, we model this albedo change as a function of soil colour, recognizing that the albedo change from crop residue will be smaller over brighter soils than darker soils. We also aim to assess whether applying a more conservative approach of the biophysical effects of conservation agriculture within an ESM can improve the simulation of present‐day climate and we explore the possible climate sensitivity to different conservation agriculture estimates.

## 2 MATERIALS AND METHODS

### 2.1 Model description and setup

We use the Community Earth System Model (CESM) version 1.2 (Hurrell, Holland, & Gent, 2013) with prescribed sea surface temperatures (SSTs) and sea ice fraction using a setup that closely follows the framework of the Atmospheric Model Intercomparison Project (AMIP). For all simulations we use the F1850PDC5 component set for the present‐day period commencing from 1976 to 2010. This includes using the Community Atmosphere Model version 5 (Neale et al., 2012) with transient greenhouse gas concentrations prescribed from measurements and the Community Land Model (CLM) version 4 (Oleson, Lawrence, & Bonan, 2010) with prescribed vegetation phenology derived from MODIS (Lawrence & Chase, 2007). The land surface heterogeneity is represented at the subgrid scale by defining multiple land units consisting of different surface types (e.g. vegetated, urban, wetland, lake, and glacier). The vegetated land unit includes 16 plant functional types (PFT) with the global distribution set to the year 2000 based on MODIS data and on Ramankutty, Evan, Monfreda, and Foley (2008) for croplands (Lawrence & Chase, 2007).

We use the same set of control simulations as those evaluated in Thiery et al. (2017). This consists of a 5‐member control ensemble with a horizontal resolution of 0.9° latitude × 1.25° longitude, starting from 1976 to 2010 (35 years), where the first 5 years are discarded as spin‐up. Ensemble members are generated by applying a random perturbation of 10^−14^ to the atmospheric temperature initial conditions (Fischer, Beyerle, & Knutti, 2013). We prescribe SSTs and sea ice to focus on the influence of land–atmosphere interactions without the added complexity of ocean–atmosphere feedbacks on the climate system. In addition to the control ensemble, we run four 5‐member ensemble experiments corresponding to the four different conservation agriculture estimates described in the following section: BASE, LOW, HIGH, and POT.

### 2.2 Description of conservation agriculture dataset

We use the conservation agriculture dataset developed by Prestele et al. (2018) to prescribe the spatial distribution of conservation agriculture in the CESM simulations. This dataset builds on the national‐level estimates of conservation agriculture published in Kassam et al. (2015) and additional regional datasets. The aggregated estimates of conservation agriculture were downscaled to a 5‐arc‐minute regular grid using GIS‐based multi‐criteria analysis. The downscaling algorithm considers several spatial determinants of conservation agriculture adoption, including biophysical (aridity and soil degradation) and socio‐economic (farm size, access to suitable equipment, and poverty level) variables. Uncertainties due to inconsistencies in the definition of conservation agriculture (e.g. Carmona et al., 2015; Hobbs, 2007) and the lack of systematic reporting schemes (Kassam et al., 2015) are represented by a range of spatially explicit maps (BASE, LOW, and HIGH). In particular, the baseline estimate (BASE) represents the most likely distribution of conservation agriculture based on the available data sources with a global area of 158 Mha. The uncertainty range in the LOW and HIGH estimates was derived from alternative country‐level datasets on tillage practices not used in the BASE estimate. Alternative data could be obtained for approximately 30% of the global arable land area. For the remaining area a default uncertainty range of ±25% was added to the national level CA areas of the BASE estimate. Globally, this resulted in 122 and 215 Mha of conservation agriculture for the LOW and HIGH estimates, respectively. To identify the global future potential for conservation agriculture, Prestele et al. (2018) also provide a POTential estimate. This estimate assumes that all agricultural land that is generally suitable for management under the principles of conservation agriculture is converted in the future (1130 Mha globally). This estimate still deviates strongly from earlier idealized implementations of conservation agriculture on all arable land because there are many conditions that constrain the adoption of conservation agriculture. For methodological details and additional information on the different estimates we refer to Prestele et al. (2018).

### 2.3 Implementation of conservation agriculture

**Figure 1:**
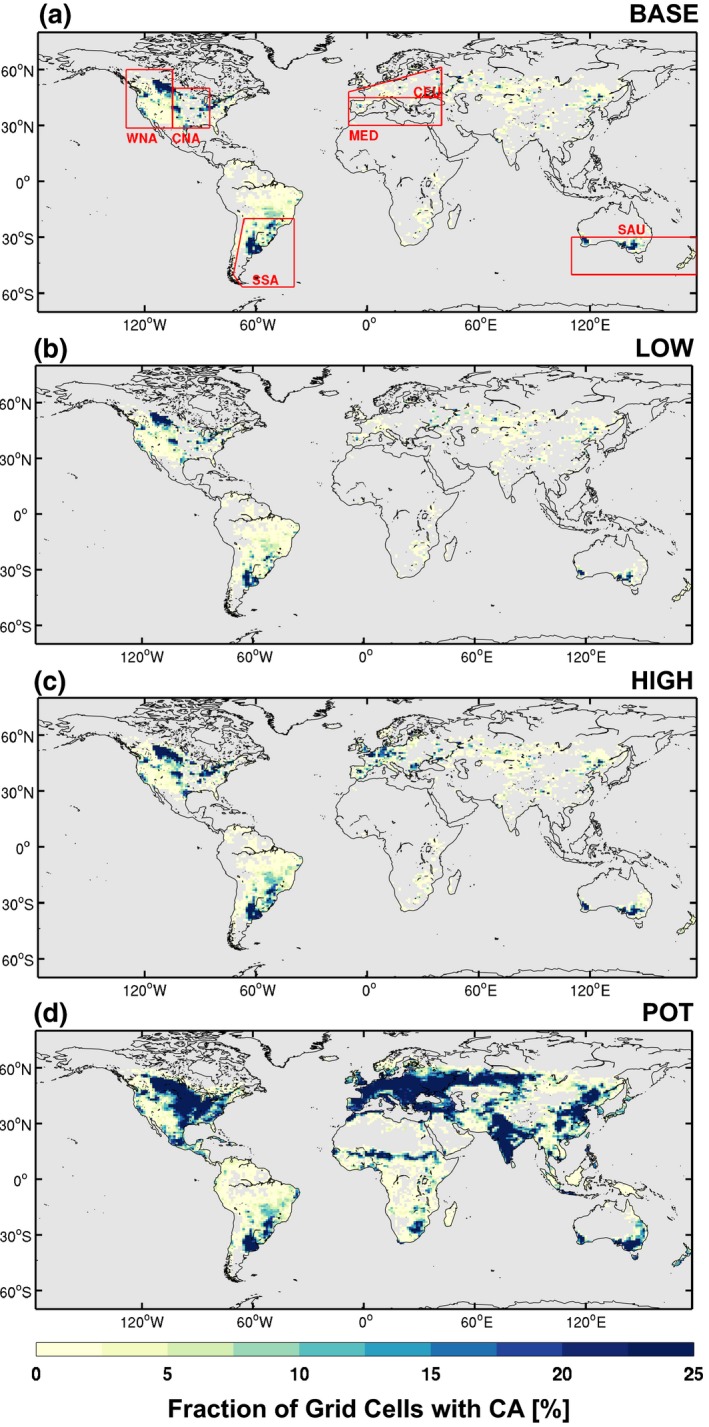
Conservation agriculture extents mapped to CLM crop PFT for the four different estimates: (a) BASE, (b) LOW, (c) HIGH, and (d) POT. The red boxes in (a) denote the regional domains examined in greater detail including Western North America (WNA), Central North America (CNA), South‐eastern South America (SSA), Central Europe (CEU), Mediterranean (MED), and Southern Australia (SAU)

We implement conservation agriculture (CA) into CESM by splitting the existing CLM crop PFT into a fraction under conservation agriculture (*C*_CA_) and a fraction under conventional management (*C*_CM_). Therefore, both forms of management are possible within a grid cell. The fractions of cropland under conservation agriculture and under conventional management are determined as follows:

(1) $$C_{\mathrm{CA}}=C_{\mathrm{ALL}}\times \left(\frac{A_{\mathrm{CA}}}{A_{\mathrm{CROP}}}\right)$$

(2) $$C_{\mathrm{CM}}=C_{\mathrm{ALL}}\times \left(1-\frac{A_{\mathrm{CA}}}{A_{\mathrm{CROP}}}\right)$$

where *C*_ALL_ is the default CLM crop fraction, *A*_CA_ is the area under conservation agriculture, and *A*_CROP_ is the total cropland area. Both *A*_CA_ and *A*_CROP_ are obtained from the CA dataset (see previous section) and are conservatively aggregated from the original 5‐arc‐minute resolution to the CLM resolution used in this study (0.9° latitude × 1.25° longitude). By using this approach we avoid potential grid conflicts, as the CA dataset is based on the HYDE cropland extent for the year 2012, whereas the CLM land cover uses 2000 cropland extents from Ramankutty et al. (2008). The distribution of the CA crop PFT for the four CA estimates is illustrated in Figure 1.

Surface albedo (*α*) in CLM is calculated at the subgrid level for canopy and soil surfaces separately, which are then aggregated to a total surface albedo as a weighted combination of snow‐free and snow‐covered albedos. All albedo terms are modelled using a two‐stream approximation for radiative transfer to determine the direct and diffuse radiation contributions. To reflect the higher surface albedo of crop residue, we increase the soil albedo for the CA crop PFT using the following function:

(3) $$\Delta \alpha_{\mathrm{soils},s}=\text{min}\;\left(0.1,\;\frac{0.1}{N_{S}\;+\;1-s}\right)$$

where *N*_*S*_ is the number of soil classes (here we use 20) and *s* is the soil colour index (1–20 with 1 the brightest and 20 the darkest soil). We limit the maximum change in soil albedo to 0.1, which is considered the maximum possible change in surface albedo by crop residue (Davin et al., 2014; Hirsch et al., 2017). Therefore, we constrain the albedo change by the soil colour, recognizing that the effective albedo change will be minimal on brighter soils compared to darker soils with the albedo change ranging from 0.005 for the brightest soil to 0.100 for the darkest soil. We assume that the crop residue is present all year, but our implementation ensures that the effect of the increased soil albedo on the total surface albedo is dampened during the growing season by the presence of canopy cover.

The presence of crop residue also has an impact on the amount of soil evaporation. Therefore, to reduce the soil evaporation, and mimic the effect of a crop residue layer for no tillage areas, we double the litter resistance of the soil column corresponding to the CA crop PFT. Note that the litter resistance is added to the aerodynamic resistance which together limit water vapour transfer from the ground to the atmosphere by scaling the soil latent heat flux. Various perturbations of the litter resistance were tested in offline land surface only simulations. We chose to double the litter resistance term so that the change in soil moisture was limited to within 10%–20%, which is consistent with observational evidence of the effect of crop residue compared to tilled soils (De Vita, Di Paolo, Fecondo, Di Fonzo, & Pisante, 2007). Furthermore, we note that the CA and conventionally managed crop PFTs have been put on separate soil columns to account for the fact that the presence of a crop residue will influence the available soil moisture of the CA crop PFT.

Due to a lack of global datasets that characterize tillage intensity, we do not implement partial residue cover. Furthermore, we do not modify the surface roughness or infiltration rates due to limitations in how these vary according to residue thickness. We restrict our implementation to changing the biophysical properties of the CA crop, but recognize that the presence of crop residues and absence of soil disturbance do influence the carbon stores in litter and the upper soil layers.

### 2.4 Evaluation datasets

We evaluate the performance of all experiments by comparing the model output to several observational datasets. For surface albedo we use the monthly European Space Agency Global Albedo product version D17 over 1998–2010 (GlobAlbedo; Danne, Zuehlke, & Krämer, 2011). For 2 m air temperature and precipitation we use the monthly Climate Research Unit version 3.22 dataset (CRU; Harris, Jones, Osborn, & Lister, 2014). We also use the monthly Global Precipitation Climatology Project version 2.2 dataset (GPCP; Huffman et al., 2001). Surface shortwave and longwave radiation is compared to the satellite‐based monthly Global Energy and Water Cycle Exchanges (GEWEX) Project Surface Radiation Budget version REL3 over 1984–2007 (SRB; Stackhouse et al., 2011). For the sensible and latent heat fluxes, we use data from the monthly Fluxnet Model Tree Ensembles dataset over 1982–2010 (MTE; Jung et al., 2010), the monthly benchmark synthesis for diagnostic evapotranspiration over 1989–2005 (LandFlux‐EVAL; Mueller et al., 2013), and the daily Global Land Evaporation Amsterdam Model version 3a (GLEAM; Miralles et al., 2011). Finally, we evaluate temperature extremes using the monthly Global Historical Climatology Network (GHCNDEX; Donat et al., 2013a) and version 2 of the monthly Hadley Centre Extremes dataset (HadEX2; Donat et al., 2013b). We conduct the model evaluation over 1981–2010 or otherwise limited to the period of data availability noted above. All observational products are regridded to the CESM resolution using conservative remapping.

### 2.5 Analysis

Our analysis uses the ensemble average daily output from CESM to examine changes in the extremes indices as defined by the Expert Team on Climate Change Detection and Indices (ETCCDI, Zhang et al., 2011). In this study we present the impact of CA on the following two extremes indices: the annual maximum daytime temperature (TXx) and the annual minimum night‐time temperature (TNn). Other ETCCDI extremes indices including the number of consecutive dry days and extreme precipitation indices did not show systematic responses to the modifications we applied in CESM and are therefore omitted.

As CESM can provide output at the PFT level we are able to examine how the distinction between the CA and CM crops influences the subgrid‐scale climate. Furthermore, we can also compare this to the standard grid‐scale surface variables (e.g. 2‐m air temperature, latent heat flux, etc.) that are calculated as a weighted combination of the fractional contributions from each of the PFTs. To evaluate the impact of CA on the grid‐scale surface climate we compute the mean difference between the experiment and the control using the ensemble mean monthly time series. We scale this mean difference by the standard deviation, to account for the internal variability in CESM such that differences that exceed the standard deviation are a real signal and not part of the noise. To examine the impact of CA at the subgrid‐scale surface climate we compute the mean difference between the CA and CM PFT‐level output, also scaled by the standard deviation to enable comparison to the grid‐scale surface climate response.

To examine changes in surface temperature (*T*_*S*_) in response to conservation agriculture, we use the surface energy balance decomposition method applied in previous studies (e.g. Akkermans, Thiery, & Van Lipzig, 2014; Hirsch et al., 2017; Luyssaert et al., 2014; Thiery et al., 2015, 2017). Here, we express the surface energy balance as:

(4) $$\varepsilon \sigma T_{S}^{4}=\left(1-\alpha \right){\text{SW}}_{i}\;+\;{\text{LW}}_{i}-\text{LHF}-\text{SHF}-R$$

where ε is the surface emissivity, *σ* is the Stefan‐Boltzmann constant (5.67 × 10^−8^ W m^−2^ K^−4^), *α* is the surface albedo, SW_*i*_ is the incoming shortwave radiation, and LW_*i*_ is the incoming longwave radiation, LHF is the latent heat flux and SHF is the sensible heat flux. The residual term (R) includes the ground heat flux and changes in subsurface heat storage. The change between the experiment and control (denoted as ∆) is calculated by taking the derivative of Equation (4) with respect to *T*_*S*_ and solving for ∆*T*_*S*_:

(5) $$\begin{array}{c} \Delta T_{S}=\frac{1}{4\varepsilon \sigma T_{S,\mathrm{CTL}}^{3}}\left(\right.-{\mathrm{SW}}_{i}\Delta \alpha +\left(1-\alpha \right)\Delta {\mathrm{SW}}_{i}\\ \;\;+\Delta {\mathrm{LW}}_{i}-\Delta \mathrm{LHF}-\Delta \mathrm{SHF}-\Delta R\left.\right) \end{array}.$$

To examine regional changes we focus our analysis over regions where conservation agriculture is extensive. We use the regions defined in the IPCC Special Report on Managing the Risks of Extreme Events (SREX) (IPCC, 2012; Seneviratne et al., 2012). Of these SREX regions, we present the results for: Western North America (WNA), Central North America (CNA), South‐eastern South America (SSA), Central Europe (CEU), Mediterranean (MED), and Southern Australia (SAU). These regions are illustrated in Figure 1a.

## 3 RESULTS

### 3.1 Sensitivity of model skill to the inclusion of CA

**Figure 2:**
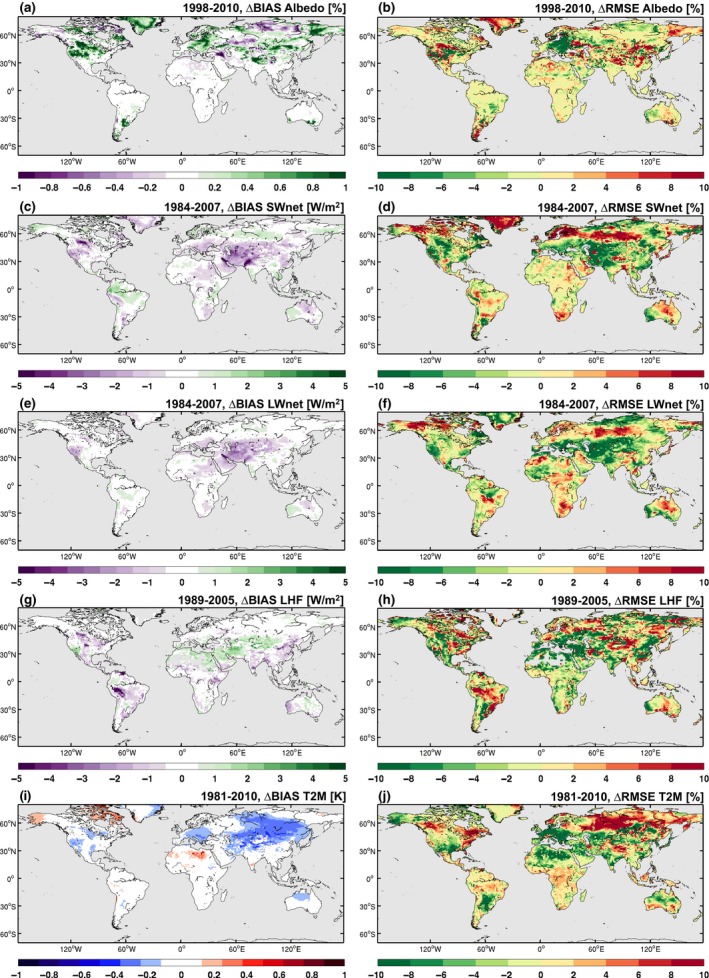
Contour maps illustrating skill changes from the implementation of conservation agriculture: (a, c, e, g, and i) change in the climatological model bias (model minus observations) between BASE and CTL and (b, d, f, h, and j) percentage change in the temporal root mean square error between BASE and CTL. Computed using the monthly time series of the ensemble means. Displayed variables are (a, b) surface albedo, (c, d) net shortwave radiation (SW_net_), (e, f) net longwave radiation (LW_net_), (g, h) latent heat flux (LHF LandFlux), and (i, j) 2 m air temperature (T2M)

In this section, we focus on how the existing CESM climate simulation skill (i.e. how well the simulations agree with the observations) is changed by including the biophysical effects of CA from the BASE experiment relative to the skill obtained in the control simulation without CA (CTL). This includes examining the change in the bias for the present‐day climatology and root mean square error (RMSE) for key variables, such as the surface albedo, net shortwave (SW_net_) and longwave (LW_net_) radiation, the latent heat flux (LHF), and 2 m air temperature (T2M) (Figure 2). While the bias in the surface albedo generally increases in the BASE experiment relative to the CTL for locations where CA is applied, this increase is very small (Figure 2a). The corresponding response in the radiation balance indicates a general decrease in the bias over large areas, particularly over Asia and North America, for SW_net_ (Figure 2c), LW_net_ (Figure 2e), and T2M (Figure 2i). For South America, there is an increase in the SW_net_ bias (~1 W/m^2^), but a decrease in the LHF bias (Figures 2c,g). Note that these changes in bias extend beyond the regions where CA is applied and, therefore, despite changes in the bias indicating some improvement in the simulation skill this result may still be influenced by the internal variability in the model. Similarly, the percent change in RMSE indicates that there is some skill improvement in simulating the temporal variability, particularly for the northern mid‐latitude regions with a 5%–10% error reduction for the surface albedo, SW_net_, LW_net_, LHF, and T2M (Figure 2b,d,f,h,j). However, at higher latitudes (50N‐90N) there is a decrease in skill with a 6%–10% increase in RMSE. Simulation skill over South‐eastern South America (SSA) also improves for T2M with a 10% reduction in error, despite some decrease in skill for LHF. Generally, Figure 2 demonstrates that there is improvement in simulation skill, but that this is not uniform across the global domain.

**Figure 3:**
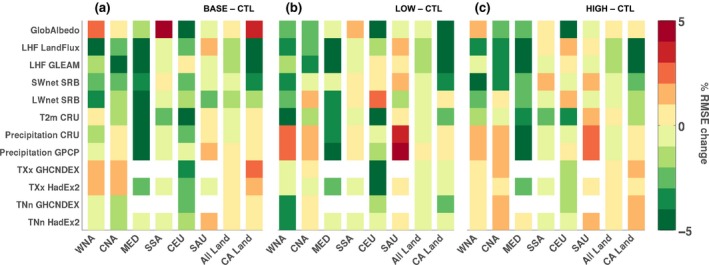
Added value of including conservation agriculture. Percentage change in the spatiotemporal root‐mean‐square error (RMSE) for (a) BASE, (b) LOW, and (c) HIGH relative to the CTL ensemble over different regions (*x‐*axis) and with respect to 12 observational products (*y‐*axis). Considered regions are the six SREX regions denoted in Figure 1 plus global land and global CA land. Observational products are for albedo (GlobAlbedo), latent heat flux LHF (LandFlux‐EVAL, GLEAM), surface net shortwave SW_net_ and longwave LW_net_ radiation (SRB), near‐surface air temperature *T*_mean_ (CRU), precipitation (CRU and GPCP), monthly maximum daytime temperature TXx (GHCNDEX and HadEX2), and monthly minimum night‐time temperature TNn (GHCNDEX and HadEX2). The RMSEs are computed for the ensemble monthly mean time series in every pixel, and subsequently averaged over the selected region. Regions with an observational coverage below 50% are marked in white

As we are interested in how the uncertainty between the different CA estimates influences simulation skill, we examine the added value of including CA for different climate variables over the regions where the CA extent is greatest (Figure 3). We evaluate the added value by calculating for each region the change (experiment minus control) in the spatiotemporal root mean square error. Accounting for CA generally improves the simulation skill over the Mediterranean for all variables and CA estimates. For other regions, including WNA, CNA, and CEU, we find enhanced skill for some variables. For SSA and SAU, the added value is limited for all CA estimates. The BASE (Figure 3a) and LOW (Figure 3b) CA estimates contribute the most to improved simulation skill, likely due to the more realistic distribution of CA in these cases. Finally, if we consider the grid cells where land fraction within CESM exceeds 50% (“all land”) or just the grid cells that have a nonzero CA fraction (“CA Land”) is present, there is added value for most variables over the grid cells where CA has been applied, particularly for the LOW estimate. Note that for precipitation the added value is sensitive to which observational precipitation product is used and is likely a result of the considerable uncertainty between these datasets (e.g. Adler, Kidd, Petty, Morissey, & Goodman, 2001). Therefore, we are confident that our implementation of the biophysical effects of CA on the regional‐scale climate has added value.

### 3.2 Effect of conservation agriculture on climate

**Figure 4:**
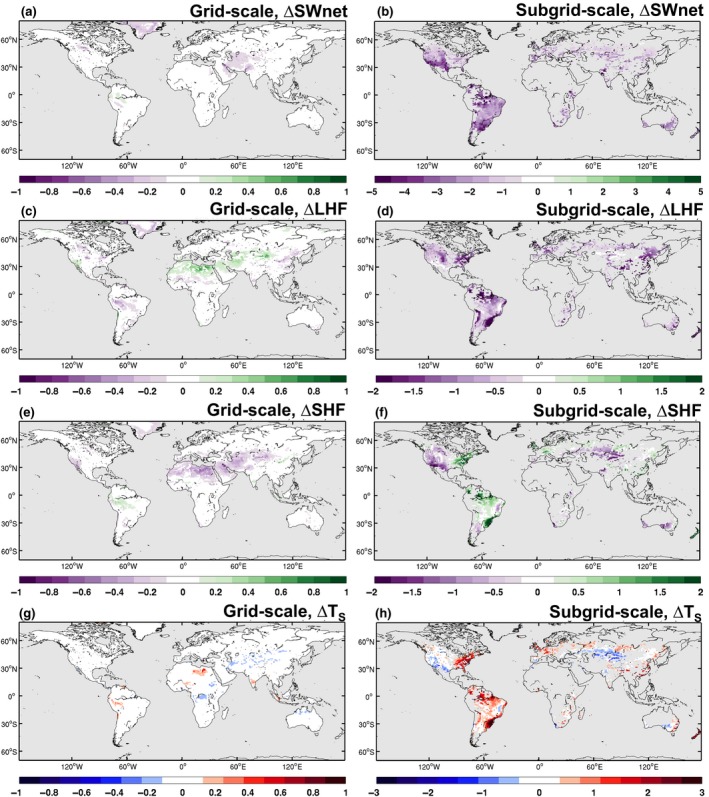
Grid‐scale (BASE minus CTL) (a, c, e, and g) and subgrid‐scale (CA(BASE) minus CM(BASE)) (b, d, f, and h) response to implementation of conservation agriculture calculated as the mean difference normalized by the standard deviation of the differences to filter out the effects of internal variability. Calculated using the monthly mean time series of all ensemble members. For (a, b) net shortwave radiation (SW_net_), (c, d) latent heat flux (LHF), (e, f) sensible heat flux (SHF), and (g, h) near‐surface temperature (*T*_*S*_). Values exceeding 1 indicate that the response exceeds the internal climate variability of CESM. Note the different colour axis limits between the grid‐scale and subgrid‐scale panels

Using the PFT‐level outputs from CLM it is possible to examine the subgrid‐scale differences between the CA and conventionally managed (CM) crops (Figure 4). This subgrid‐scale effect (CA minus CM), representing the local effect of CA, can be compared to the “grid scale” effect computed by comparing the BASE and CTL simulations. To remove the internal climate variability from the signal we normalize the mean difference, either BASE minus CTL for grid scale or CA minus CM for subgrid scale, by the standard deviation. Generally, at the grid scale, changes in SW_net_, LHF, SHF, and *T*_*s*_ are small (Figure 4, left column), suggesting that the model response is dampened by internal climate variability. However, the subgrid‐scale response (Figure 4, right column), which shows the difference between the CA and CM crops for the BASE estimate, is substantially larger and often exceeds the internal climate variability of CESM.

In particular, the subgrid‐scale responses provide insight into how CA influences the local‐scale climate. More specifically, decreases in SW_net_ (Figure 4b) are consistent with the increase in background albedo and tend to be related to the CA fraction with larger decreases in North and South America. The decrease in energy absorbed at the surface contributes to the decrease in LHF (Figure 4d), which is further amplified by the increased resistance to soil evaporation due to the presence of the crop residue. Again this response is larger over regions where the CA fraction is greater. The SHF response is not always consistent with the decrease in SW_net_, with some increases over Eastern North America and South‐eastern South America. However, this is likely due to the change in the partitioning between LHF and SHF arising from the increased resistance to soil evaporation. These changes in SHF explain why some regions result in warming of *T*_*s*_ (Figure 4h) rather than cooling as may be expected by reducing the amount of shortwave energy absorbed by the surface.

**Figure 5:**
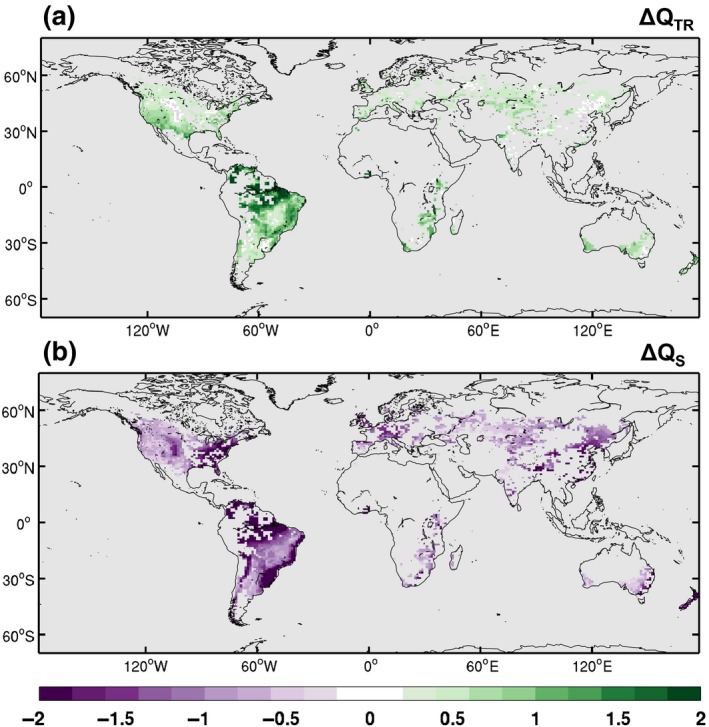
Subgrid‐scale response to implementation of conservation agriculture for (a) canopy transpiration (Q_TR_) and (b) soil evaporation (Q_S_) for the BASE CA estimate. The response is calculated as the mean difference (CA minus CM) normalized by the standard deviation of the differences to filter out the effects of internal variability. Calculated using the monthly mean time series of all ensemble members. Values exceeding 1 indicate that the response exceeds the internal climate variability of CESM

At the subgrid scale, the presence of the crop residue also influences the partitioning of the LHF between plant transpiration (Figure 5a) and soil evaporation (Figure 5b). Coincidently, the warming from CA (e.g. Figure 4h for *T*_*s*_) often occurs when the decrease in soil evaporation exceeds the increase in canopy transpiration (e.g. South America and Eastern North America; Figure 5). Cooling occurs when the decreased soil evaporation is comparable to the increase in transpiration. Increases in transpiration arise from more moisture availability due to less soil evaporation, and therefore, the thickness of the crop residue is likely to have some influence modulating local temperatures.

Note that similar responses were found for the LOW and HIGH CA estimates for the climate variables presented in Figures 4 and 5 with the exception of POT where the application of CA is much greater and therefore, the corresponding subgrid‐scale changes between the CA and CM crops were larger over Europe and Asia.

**Figure 6:**
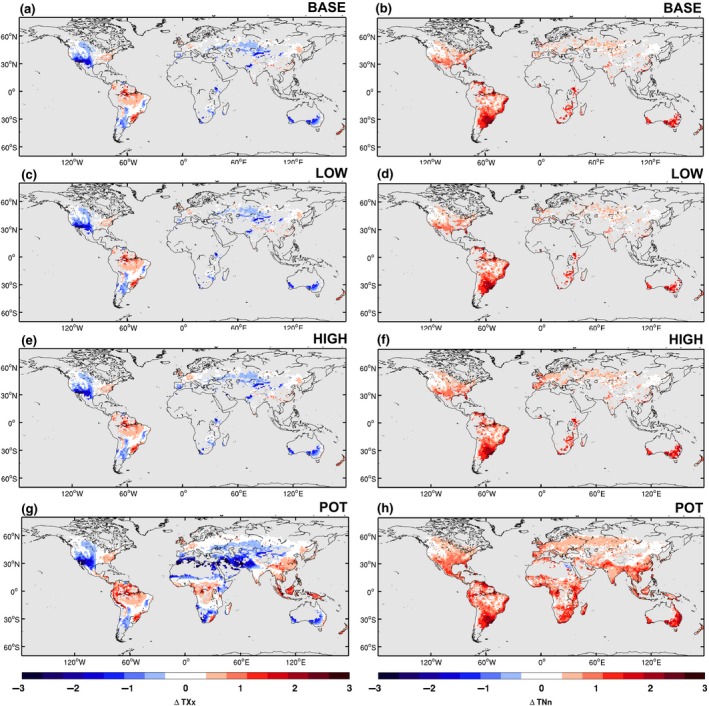
Subgrid‐scale response to implementation of conservation agriculture for the annual maximum daytime temperature (TXx; a, c, e, and g) and the annual minimum night‐time temperature (TNn; b, d, f, and h) for the different CA estimates: (a, b) BASE, (c, d) LOW, (e, f) HIGH, and (g, h) POT. The response is calculated as the mean difference (CA minus CM) normalized by the standard deviation of the differences to filter out the effects of internal variability. Calculated using the monthly mean time series of all ensemble members. Values exceeding 1 indicate that the response exceeds the internal climate variability of CESM

In the literature possible cooling of hot temperature extremes has been found for regions where tillage is limited (e.g. Davin et al., 2014). Therefore, we examine the subgrid‐scale changes in both the annual maximum daytime 2 m air temperature (TXx) and the annual minimum night‐time temperature (TNn) for each of the CA estimates (Figure 6). Generally, CA induces cooling of TXx by more than 1°C for most regions where CA is applied for all estimates (Figure 6, left column). Interestingly, TXx increases by 1°C over Amazonia in South America where CA is not practiced. In contrast, CA leads to warming of TNn consistently of 1°C or more, with the greatest warming occurring in the locations where CA fraction is greatest in the Southern Hemisphere: South‐eastern South America, South Africa and Southern Australia. Note that the response to TNn is larger in magnitude than the response in TXx. This is likely due to how we parameterize CA, where the leaf area index (LAI) modulates how much of the soil surface is exposed. A larger LAI would therefore lead to a smaller contrast between the CA and CM crops, which coincides with TXx during the summer season. The subgrid‐scale response of TXx and TNn is also remarkably similar between the BASE, LOW, and HIGH CA estimates (Figure 6a–f), indicating that the sensitivity of CESM to these different CA estimates is low at this resolution.

### 3.3 Effect of conservation agriculture on the surface energy balance

**Figure 7:**
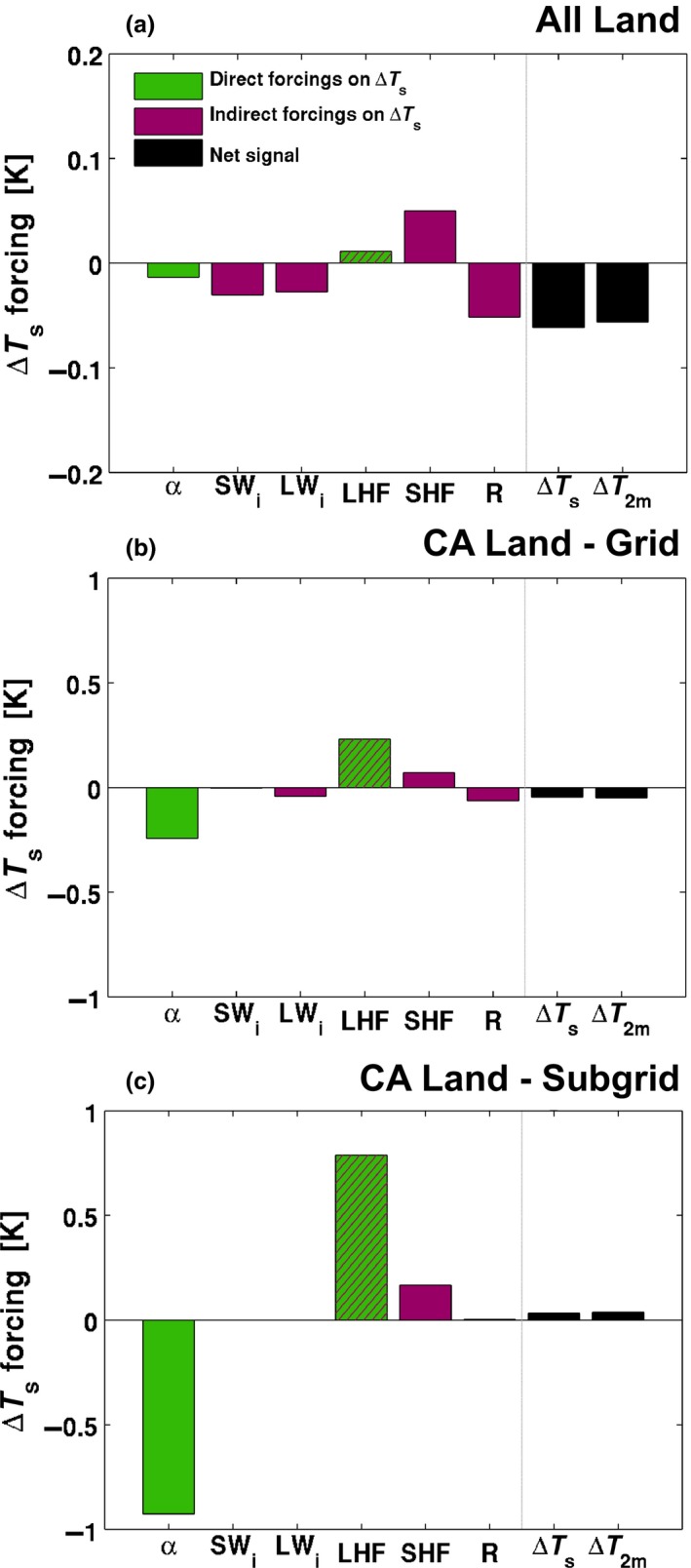
Changes in surface temperatures explained by changes in the surface energy balance from conservation agriculture; comparison between BASE and CTL ensemble climatological means. Individual direct (green), indirect (purple), and mixed (hatched) forcings to Δ*T*_*s*_ as described in Equation (5) over (a) all land, (b) CA land, and (c) CA land—subgrid effect (all units are in K). Along the *x*‐axis: *α* denotes the change in *T*_*s*_ caused by a modified albedo, SW_*i*_ by changing incoming shortwave radiation, LW_*i*_ by changing incoming longwave radiation, LHF by changing evapotranspiration, SHF by changing sensible heat flux and R by changes in other components (subsurface heat flux and anthropogenic heat fluxes). The impact on *T*_2m_ is also shown for reference. Note the different *y*‐axis scales

**Figure 8:**
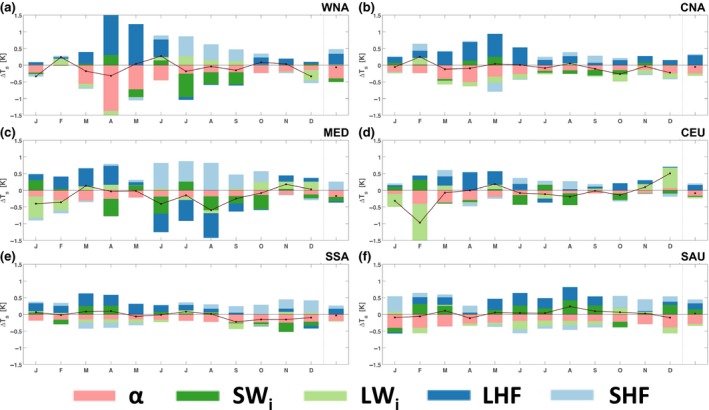
Seasonal cycle of changes in surface temperature explained by subgrid changes in the surface energy balance due to conservation agriculture for the BASE CA estimate using the climatological monthly ensemble mean. Monthly mean contributions to Δ*T*_*s*_ as described in Equation (5) over the managed pixels of Western North America (WNA; a), Central North America (CNA; b), South Europe and Mediterranean (MED; c), Central Europe (CEU; d), South‐eastern South America (SSA; e), and Southern Australia (SAU; f). The individual contributions are visualized as stacked bars, whereas the black line and dots show the net *T*_*s*_ change. The impact on annual mean *T*_*s*_ is shown right of the grey line. Legend: *α* = pink, SW_*i*_ = dark green, LW_*i*_ = light green, LHF = dark blue, SHF = light blue

The response of both the grid‐scale and subgrid‐scale climate to the implementation of CA in CESM indicates that there are two main competing effects that influence the surface temperature response. This includes (a) changes in the surface albedo, which alters how much energy is available to be partitioned between the sensible and latent heat fluxes, and (b) changes in the surface resistance which influences the partitioning between these fluxes. Therefore, an examination of the change in surface temperature due to the surface energy balance response is warranted at both global (Figure 7) and regional (Figure 8) scales.

At the global scale the net effect of CA on all global land grid cells is a cooling (within 0.1°C) which is largely attributed to decreases in incoming radiation (SW_*i*_ and LW_*i*_) and enhanced residual heat storage (Figure 7a) despite changes in SHF contributing to warming. If one considers the CA grid cells only, then the net effect on surface temperature is still cooling (within 0.1°C, Figure 7b, note different axis limits). However in this case, this is largely attributed to the change in surface albedo, which is larger than the changes in LHF that lead to warming. Finally, at the subgrid scale (Figure 7c) it is the albedo and LHF changes that dominate the temperature response, with the influence of the SHF contributing to a slight increase in surface temperature. The contrast between Figure 7a and Figures 7b and 7c indicates that the introduction of CA may have larger‐scale impacts on the climate that may be a model dependent result. Previous research with CESM examining albedo enhancement (Hirsch et al., 2017) found that this model has a tendency to produce large cloud feedbacks when more energy is reflected at the surface. Given that Figure 7a includes all land grid cells, the effective area that the albedo and LHF change from CA influences temperatures is relatively smaller. Instead, the cloud feedbacks that propagate from the introduction of CA have a larger nonlocal influence reflecting the larger contribution of SW_*i*_ and LW_*i*_ on changes in surface temperature. Note that in Figure 7 we distinguish between the direct forcings (i.e. albedo change) and the indirect forcings (i.e. SWi, LWi, and SHF), which are modified indirectly by land–atmosphere feedbacks.

As evident in Figure 6, this global aggregation of the surface energy balance decomposition of surface temperature is likely to mask regional and seasonal differences and, therefore, we focus on the monthly time‐scale changes for six regions (denoted in Figure 1) where the CA fraction is greatest (Figure 8). Here, we just show the results for the BASE experiment. Overall, some regions show more seasonality in the surface energy balance controls on surface temperature (*T*_*s*_) than others (e.g. WNA, CNA, MED, and CEU). For example, the change in *T*_*s*_ is predominantly influenced by changes in LHF and albedo in MAM for WNA and CNA, however, in JJA the SHF has a larger influence for the WNA and MED regions. For MED and CEU, LW_*i*_ has a larger influence on *T*_*s*_ during DJF. For the SSA and SAU regions, the net change in *T*_*s*_ is often zero, or close to zero, for most months of the year and may be associated with the opposing influences of albedo and LHF change. There are, however, instances where the net change in *T*_*s*_ is nonzero and this often occurs when changes in LW_*i*_, SHF, and SW_*i*_ are larger. We note that there was limited sensitivity in the surface energy balance decomposition between the LOW and HIGH CA estimates (not shown), perhaps associated with the resolution of our simulations where differences in CA extent between the estimates are less resolved. For the POT estimate (not shown) the changes in *T*_*s*_ due to individual fluxes tend to be larger than the more conservative CA estimates. However, this does not appear to shift the seasonality of the response, despite the potential for land–atmosphere feedbacks to enhance the response. Overall, the presence of CA has a considerable impact on the surface energy balance at the subgrid scale.

**Figure 9:**
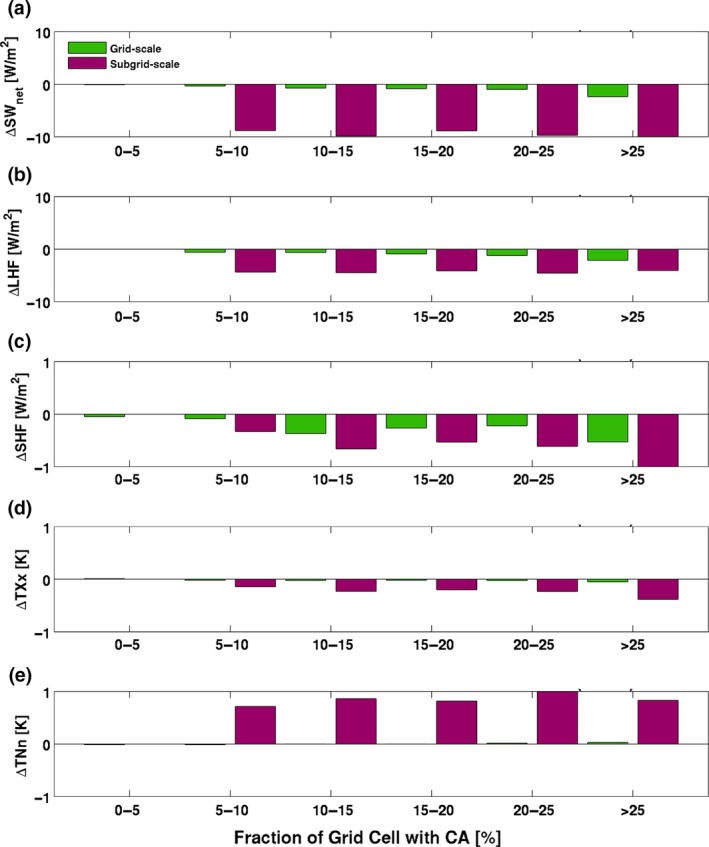
Histograms comparing the grid‐scale (BASE minus CTL) and subgrid‐scale (CA(BASE) minus CM(BASE)) response to the implementation scale of conservation agriculture. Calculated using monthly mean time series of all ensemble members. For (a) net shortwave radiation (SW_net_, W/m^2^), (b) latent heat flux (LHF, W/m^2^), (c) sensible heat flux (SHF, W/m^2^), (d) annual maximum daytime temperature (TXx, K), and (e) annual minimum night‐time temperature (TNn, K)

Changes in the surface energy balance at the subgrid scale are a function of the CA fraction, which also influences the extent to which the subgrid‐scale response influences the grid‐scale response (Figure 9). Generally, for SW_net_, LHF, and SHF (Figure 9a–c), there is an approximate linear increase in the change in these energy fluxes with the percentage of the grid cell with CA. In particular, the grid‐scale change in these energy fluxes also shows a general linear relationship, although the changes are at a much smaller magnitude than the subgrid‐scale response, consistent with the results shown in Figure 4. The grid‐scale response is generally negligible when examining changes between the BASE and CTL ensembles for the 2 m air temperature extremes TXx (Figure 9d) and TNn (Figure 9e), despite considerable changes at the subgrid scale. Nonetheless, Figure 9 demonstrates that the CA fraction does influence how much the subgrid‐scale distinction between CA and CM crops can influence the grid‐scale climate, particularly when the extent exceeds at least 25% of the grid cell. This suggests that representing CA in the land surface component of an ESM does have some resolution dependence and therefore is highly relevant for simulating local‐ to regional‐scale climates.

### 3.4 Future biogeophysical mitigation potential of CA

In this section, we investigate the biogeophysical impact of a future scenario of large‐scale conversion to CA. The POT experiment represents the present‐day climate where all croplands suitable for CA are effectively converted. The purely local effect of CA in the POT experiment (i.e. subgrid‐scale effect under the atmospheric forcing) is a strong cooling of TXx (Figure 6g). Only some regions such as the Amazon, Africa, and Indonesia show a warming response to CA. This is likely due to changes in evapotranspiration dominating over the changes in albedo, which have been found in previous land use change experiments to occur in tropical regions (Davin & de Noblet‐Ducoudré, 2010) in addition to the presence of the crop residue changing the partitioning of the LHF between soil evaporation and transpiration (e.g. Figure 5). The local cooling of TXx often exceeds 1°C, reaching 3°C over the locations where the CA expansion is greatest. These decreases in TXx are comparable in magnitude to the projected warming with climate change found for CESM in Hirsch et al. (2017) and more generally for CMIP5 in Seneviratne, Donat, Pitman, Knutti, and Wilby (2016). These results suggest that a large‐scale roll out of CA may locally offset part of the projected future warming of TXx in some regions. However, increases in TNn are often larger in magnitude. This may present a limitation to promote the expansion of CA into new areas.

**Figure 10:**
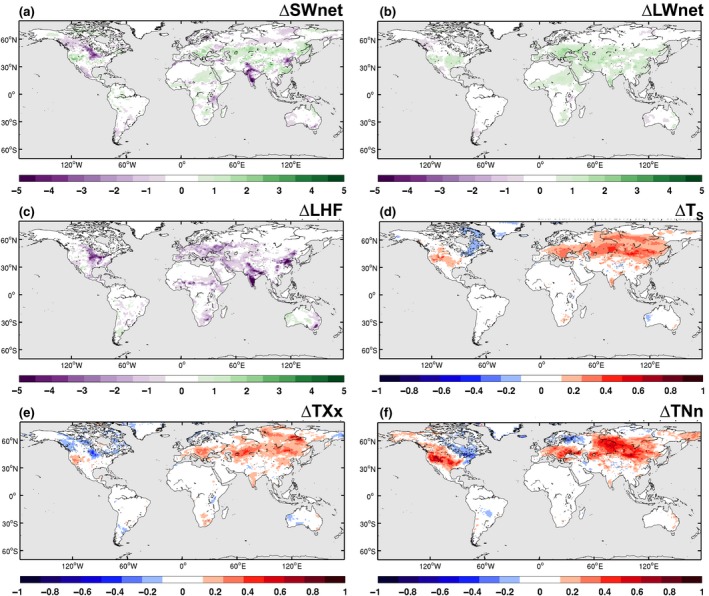
Contour maps illustrating the grid‐scale difference between the POT and BASE experiments. Calculated from the monthly time series of all ensemble members. For (a) net shortwave radiation (SW_net_; W/m^2^), (b) net longwave radiation (LW_net_; W/m^2^), (c) latent heat flux (LHF; W/m^2^), (d) surface temperature (*T*_*S*_; K), (e) the annual maximum daytime temperature (TXx; K), and (f) the annual minimum night‐time temperature (TNn; K)

We also examine the total mitigation potential of CA by comparing the grid‐scale climate in the POT experiment to the BASE experiment (Figure 10). Here, the difference in available energy (e.g. SW_net_ and LW_net_) is largely within 1 W/m^2^ except for the Northern United States and India where the difference in SW_net_ exceeds 5 W/m^2^ (Figure 10a,b). The LHF (Figure 10c) is also damped in POT with differences ranging from 1 to 5 W/m^2^ dependent on the intensity of the CA expansion. However, when comparing the grid‐scale temperature response to the BASE experiment (i.e. present‐day level of CA adoption) there is a general warming in POT (Figure 10d–f) over North America, Europe, and Asia, suggesting that CA could be a counterproductive climate mitigation measure if implemented at such a large scale. The apparent contradiction between the subgrid‐scale cooling and large‐scale warming effect of CA is due to the role of atmospheric feedbacks. The decrease in evapotranspiration, both due to higher albedo and to the higher soil resistance, triggers a decrease in cloud cover in the model that increases incoming radiation and thus temperature as seen in other studies (Hirsch et al., 2017; Wilhelm et al., 2015). However, we note that most of the differences in the grid‐scale response between POT and BASE were found to be not statistically significant and therefore requires further investigation to understand the potential for atmospheric feedbacks to negate any local cooling potential from CA.

## 4 DISCUSSION

Conservation agriculture is a form of land management that is extensively practiced in several of the major agricultural regions. In this study we present the first results of implementing a new spatially explicit global dataset of conservation agriculture (CA) within an Earth System Model (ESM) in order to more realistically assess the influence of CA on the climate. We find that including the biophysical characteristics of CA does add value to the simulation of the surface energy fluxes, where the changes are prominent at the subgrid scale. Our results show that at the subgrid scale, CA does contribute to local cooling of hot temperature extremes, however, at the grid scale, warming is observed associated with atmospheric feedbacks. There are some regions where CA contributes to local warming, due to (a) changes in the partitioning between latent and sensible heat fluxes and (b) the presence of the crop residue leading to decreases in soil evaporation that exceed the increase in canopy transpiration.

### 4.1 Discussion of CESM results

Our results demonstrate that by splitting the default crop PFT in CESM into CA and conventionally managed (CM) crops we are able to improve simulation skill of the incoming radiation components, the latent heat flux, and the 2 m air temperature. We find that the presence of CA has two competing effects on surface temperature consistent with the findings of Davin et al. (2014) who use a more idealized representation of CA. That is, the increase in surface albedo decreases the amount of energy available to heat the surface, and the presence of a crop residue limits soil evaporation which can also indirectly contribute to surface warming if the decrease in soil evaporation offsets the increase in plant transpiration (Figure 5). Note that the increase in plant transpiration is possible due to higher moisture availability in the soil for plants to access. Increasing the surface albedo generally reduces both the sensible and latent heat fluxes, due to decreased net radiation at the surface. However, by decreasing the soil evaporation more energy can be partitioned into sensible heating, which leads to warming of the near‐surface air. Cooling occurs when both the sensible and latent heat fluxes decrease. Therefore, warming tends to occur when the change in soil evaporation has the more dominant influence on the surface energy balance and cooling tends to occur where the change in albedo dominates.

A new result that is relevant to the ESM community is that the subdivision of a generic crop PFT into CA and CM crops has a substantial impact on the simulation of the subgrid‐scale climate. This does influence the simulation skill of the grid‐scale climate, particularly when the CA extent exceeds ~20%–25% of the grid cell. This suggests that regional‐scale simulations may benefit from including a more comprehensive representation of agricultural land management practices. Furthermore, the impact of CA on climate is comparable between the three CA estimates (BASE, LOW, and HIGH) in CESM. It is possible that aggregating the estimates to our CESM resolution removes the distinguishing features between them that characterize the CA uncertainty that may be further resolved at higher resolutions.

It is necessary to acknowledge the potential model dependence of our results. Should implementation occur with another ESM it is likely that this may be done differently, particularly if the land surface scheme does not characterize differences in soil colour to modulate the contrast in albedo between till and no till or include a litter resistance to emulate the effect of a crop residue on soil evaporation. The differences between ESMs are also not limited to the land surface parameterization, with atmospheric feedbacks likely to further perturb differences in the grid‐scale climate response. In particular, previous research has shown that surface albedo enhancement in CESM can produce substantial cloud feedbacks that in turn influence the local climate (Hirsch et al., 2017). Therefore, implementation of CA within a different ESM would be ideal to confirm both the grid‐scale and subgrid‐scale changes in surface climate that we report here.

One limitation of our implementation of CA within CESM is the use of MODIS phenology to prescribe the leaf and stem area indices (LAI and SAI, respectively). MODIS SAI includes litter and therefore information on the presence of crop residues. Furthermore, MODIS LAI does not adequately capture crop planting and harvesting cycles (Davin et al., 2014; Lawrence & Chase, 2007). This will influence the timing in our simulations to which soils are more exposed after crop harvest. As MODIS LAI tends to overestimate crop cover, our approach potentially dampens the effect of CA on surface climate. Running CESM with the carbon–nitrogen cycle and the prognostic crop model on to explicitly calculate LAI could partly resolve this limitation provided that the seasonality of crop phenology is constrained using existing data on plant and harvest dates (e.g. Sacks, Deryng, Foley, & Ramankutty, 2010).

### 4.2 Implications for future model development

Our results highlight the importance of improving the representation of agricultural land management within ESMs. While we have only included the biophysical characteristics of CA in our implementation; these effects already prove to be substantial. However, CA also involves distinguishing between different degrees of soil disturbance that has consequences for soil carbon sequestration, which is pertinent for climate change mitigation activities aimed at increasing the potential of carbon sequestration over agricultural regions. Furthermore, developing the parameterization of CA within CESM will need to involve carbon pool modifications by including information on how CA alters soil carbon. Including information on crop rotations would also be necessary and may be possible using a global dataset characterizing crop planting and harvest dates (Sacks et al., 2010). Furthermore, there are already several ESM groups developing the parameterization of irrigation practices due to the known impacts of irrigation on local climate. Integrating both irrigation and CA into ESMs will require data to identify regions where only irrigation, only CA or both are applied. Other data requirements include CA extent per crop type (e.g. maize and temperate cereals) to enable integration of CA within a prognostic crop model that accounts for different crop types and their growing cycles (e.g. Levis et al., 2012; Sacks et al., 2010). Finally, information characterizing the tillage percentage in terms of no‐till, minimal tillage, or conventional tillage and mulch depth would be ideal to split the managed crop further to examine the effects of, for example, soil disturbance depth and partial crop residue cover compared to the all or nothing approach examined here. Therefore, there is plenty of scope for further model development as data become available to characterize differences in crop types and their rotations (Erb et al., 2017). However, including all of these different crop types and management practices will require a more comprehensive approach to representing these individual management techniques within an ESM.

### 4.3 Implications for climate mitigation claims and outlook

Future expansion of CA is likely, with two potential scenarios for this expansion discussed in Prestele et al. (2018). By comparing the impact of a future potential CA extent to the present‐day distribution on climate, we present a first estimate of biophysical benefits and trade‐offs of this cropland management strategy (Figures 6 and 10). Our results indicate that large‐scale expansion of CA management could decrease temperature extremes over the mid and high latitudes on the local scale. There is, however, also a warming response both for mean and extreme temperatures over wide areas of the tropics, which would increase the vulnerability of CA managed systems to climate change at these locations. Indeed, further ESM‐based studies, implementing CA management, are required to confirm these responses and receive a robust signal. Moreover, next to these biophysical implications, enhanced soil carbon sequestration in CA managed land is discussed as a potential contribution to future net negative emissions (Neufeldt et al., 2015; Powlson et al., 2014; Smith et al., 2008) and have not been quantified yet at the large scale using ESMs. Developing the parameterization of CA within CESM further to include biogeochemical influences is therefore necessary. Nevertheless, our study provides an important step towards the explicit parameterization of CA management in ESMs and the quantification of related climate impacts and feedbacks.
